# Central European Sample Analysis of Traumatic Vertebral Fractures: A One-Year Retrospective Cohort Study

**DOI:** 10.3390/healthcare14081114

**Published:** 2026-04-21

**Authors:** Eleonora Colella, Hans-Christoph Pape, Ladislav Mica

**Affiliations:** Department of Traumatology, University Hospital of Zurich, 8091 Zürich, Switzerland

**Keywords:** vertebral fractures, sex differences, Hounsfield unit, computed tomography, combined injury

## Abstract

Background/Objectives: The purpose of this study was to examine the sex-specific distribution of traumatic spinal fractures and potential predictive clinical factors for a more well-founded treatment evaluation. Methods: This study is a retrospective cohort study. Data from electronic medical records were analyzed and compiled in a database. Demographic information, trauma-specific characteristics, and radiological measurements, as well as laboratory values and surgical treatments, were collected. Only surgical cases were included in this study. Statistical analyses were performed using the IBM SPSS Statistics program. Chi-square tests, effect sizes, and 95 confidence intervals were used for comparison of categorical variables, and means and standard deviations were calculated, as well as Levene’s test for equality and *t*-tests for analyzing continuous variables. The statistical significance was set at a two-tailed *p* < 0.05. Results: A total of 164 patients were included, with a mean age of 58.03 years. Statistically significant differences between sexes were found in age (*p* = 0.04), GCS (*p* = 0.03), hemoglobin (*p* = 0.03), hematocrit (*p* = 0.007), and the one-year post-surgical intervertebral angle (*p* = 0.004). AIS score showed statistically significant differences in the cervical and lumbar sections (*p* < 0.015; *p* = 0.022) and the overall spine (*p* = 0.049). No statistically significant difference in the HU values in the vertebra above the fracture was found between men and women. Women showed significantly larger one-year postoperative intervertebral angles than men. Conclusion: Vertebrae with lower HU values tend to collapse despite stable surgical treatment; therefore, additional bone quality assessment should be contemplated. These findings highlight sex-specific considerations for future clinical decision-making.

## 1. Introduction

Vertebral fractures are primarily caused by osteoporosis—a condition characterized by decreased bone mass and density. Osteoporosis most frequently causes a compression fracture at the thoracolumbar junction. The second most common cause of a vertebral fracture is traumatic injury to the spine [1].

Traumatic vertebral fractures can be characterized by their location and fracture type. Locations for traumatic fractures are subdivided by the AO Spine Classification System into occipito-cervical, subaxial cervical, thoracolumbar, and sacropelvic [2]. The type of fracture depends on the type of mechanical load causing the fracture—including axial loading, rotational forces, or dislocation injuries [1]. A prospective osteoporosis study concluded in 2002 showed the incidence of osteoporotic fractures to the vertebra in Europe to be 10.7/1000 women and 5.7/1000 men aged 50 years and over per year, with the highest rates being in Sweden. An increase in the incidence of osteoporotic vertebral fractures with age in both genders was also observed [3]. Vertebral fractures not only cause acute and chronic back pain but are also associated with an increased mortality for the patients [4], particularly in patients receiving conservative treatment [1]. This has also been shown by a Korean study, which reported comparable mortality rates after a vertebral fracture between South Korea and Switzerland [5]. Risk factors include age-related, degenerative, genetic, environmental, hormonal, or physical factors [6]. As a diagnostic method, the use of computed tomography imaging has been shown to be an ideal modality to safely evaluate fractures of the vertebra. For further investigation of the cause of the fracture, a Dual-Energy X-ray Absorptiometry (DEXA) was declared the gold standard for suspected osteoporotic fractures (only used to diagnose osteoporosis, not the fracture itself) [1].

A major issue with vertebral fractures is that a large proportion of affected patients do not show clinically relevant signs [3]. Therefore, more extensive research is necessary to enable early identification of clinically relevant cases and to prevent further occurrences—hopefully resulting in a lower mortality rate in patients with traumatic vertebral fractures.

The aim of this study was to analyze the data of 164 patients suffering from a traumatic vertebral fracture, with the intention to investigate the sex-specific distribution of traumatic spinal fractures and potential predictive clinical factors, providing hypothesis-generating observations for more well-founded treatment evaluations, including osteo-anabolic treatment and methyl methacrylate (MMA) [7].

## 2. Materials and Methods

### 2.1. Study Characteristics

This study was conducted as a retrospective single-center cohort study at the Department of Traumatology at the University Hospital Zurich. Data were obtained from the hospital’s electronic regional register over a one-year period between January 2020 and December 2020. Patient data were collected and compiled into a database between May 2024 and August 2024. To ensure the patients’ privacy, all data were pseudonymized and extracted according to predefined rules.

The review focused on the medical records of patients who presented to the Department of Traumatology in the year 2020. If patients met the inclusion criteria, radiological imaging reports were also incorporated. Patients with missing data were excluded from the respective analyses but not from the overall study, since other variables remained available. This resulted in different sample sizes between analyses.

### 2.2. Description of Database

The purpose of the database was to compile potentially relevant information to analyze associations between spinal fractures and other pre-existing factors. Additionally, the database may serve as a basis for further research and may be expanded in the future. The database was transferred into IBM SPSS Statistics for analysis. The relevant data from the Department of Traumatology’s patient information system were available, including trauma protocols, surgery reports, anesthesia reports, and blood test results, as well as radiological imaging and reports. The data were collected in accordance with Good Clinical Practice (GCP) guidelines [8].

### 2.3. Patient Sample

The patient sample consisted only of patients with isolated vertebral fractures and vertebral fractures combined with other fractures. A total of 164 patients were included in the study. Patients were screened, deemed eligible, included, and subsequently analyzed. Conservatively treated fractures were excluded.

### 2.4. Definition of Variables

The following patient characteristics were analyzed. These included age and sex. Sex was documented as a binary variable. Demographic data included the body mass index (BMI) and laboratory values obtained on the day of admission. Part of the blood panel was calcium, sodium, potassium, and C-reactive protein (CRP) prior to surgery, as well as leukocytes, hemoglobin, and hematocrit. All analyses were performed by the Institute for Clinical Chemistry of the University Hospital Zurich.

Trauma severity was classified as single trauma or combined injury. In patients with polytrauma, the traumatic brain injury was assessed using the Glasgow Coma Scale (GCS) [9]. Polytrauma was defined as the presence of at least two or more sustained injuries.

The anesthesia reports provided information on pre-existing conditions, with a particular focus on diabetes and tumors, as well as the American Society of Anesthesiologists (ASA) Physical Status [10] to assess the extent of multimorbidity. Chronic medication at the time of admission was recorded. Injury-specific variables constituted a major part of the database. Fracture location was categorized into three sections: cervical, thoracic, and lumbar. Isolated sacral fractures were excluded, as these are typically managed within hip and pelvic surgery. The fractures were classified according to the AO Spine Classification System [2]. The AO classifications were obtained from trauma or surgical reports. If fractures from more than one section of the spine occurred, an AO value was given to each section separately. Given the high prevalence of polytraumatic patients in the Department of Traumatology, the decision was made to add the number of the Abbreviated Injury Scale (AIS), as well as the injury severity score (ISS) [11]. The Abbreviated Injury Scale was subdivided into an AIS for general spine, cervical spine, thoracic spine, lumbar spine, head, thorax, abdomen, pelvis, extremities, and skin.

Hounsfield unit (HU) measurements were obtained from computed tomography (CT) scans. HU values were measured in the vertebra above and below the fracture. HU measurements were taken from the vertebral body and measured in the central midsagittal plane of the vertebra [12]. If more than one vertebra was fractured, HU values from the first fully intact vertebra above and below the broken one were used. Additional radiological measurements were performed with Deep Unity Review (version 2.0.2.2, Dedalus, Bonn, Germany). CT images were aligned in the sagittal plane, selecting the slice in which the spinosus process appeared at its maximum size, thereby ensuring analysis of the mid-vertebral axis. A centrally placed ROI (Region of Interest) was used and sized to avoid the cortical bone, therefore reducing the risk of artifacts. The measurements were not blinded, and interobserver reliability was not assessed, since the measurements were performed by a single person.

For possible long-term studies, a more outcome-based variable was included, where the intervertebral angle between the inferior endplate of the upper vertebra and the superior endplate of the lower fractured vertebra was measured. Data was obtained from preoperative, postoperative, and one-year follow-up imaging. If the patient had a pre-existing diagnosis of osteoporosis with an available and current T-score from a DEXA scan, it was also included in the database.

Additional surgical data consisted of a specific description of the surgical procedure done on the patient and the instrumentation used for the treatment. Surgical treatments were organized into six categories—spinal fusion, ventral cage, ventral plate, vertebroplasty, kyphoplasty, cement augmentation, and decompression. A single surgery could be divided into multiple procedures.

### 2.5. Ethics

This study was approved by the Cantonal Ethics Commission of Zurich, Switzerland, at the following BASEC number: 2021-00391.

### 2.6. Statistical Analysis

IBM SPSS Statistics (version 29.0.0.0) was used to perform all statistical analyses.

The significance was set at *p* < 0.05. Categorical variables were expressed as percentages and counts. Continuous variables were reported as mean value ± standard deviation (SD), and the standard error of the mean (SEM) was also calculated. A characterization of the Bayesian analysis was calculated, along with its analogous 95% confidence intervals (95% CI).

To assess the equality of variances, Levene’s test was applied, with the results reported for both equal and unequal variances. For further analysis of the statistical difference between values, effect sizes were calculated using Cohen’s d, Hedges’ g, and Glass’s ∆.

To examine the association between the categorical variables, the following chi-square tests were applied: Pearson chi-square test, continuity-corrected chi-square, likelihood ratio, and linear-by-linear association. In addition to these chi-square tests, Fisher’s exact test was used.

## 3. Results

### 3.1. Gender Comparison of Continuous Variables

The cohort included 93 (56.7%) male patients and 71 (43.3%) female patients. The mean (±SD) age of all patients was 58.03 ± 20.016 years. The mean age was 61.69 years in women and 55.23 years in men. This difference was statistically significant (*p* = 0.04; 95% CI [−12.62, −0.28]). Among male patients, 61.29% had a polytrauma, while 39.78% had isolated trauma. A total of 56.34% of women had a single trauma, and 43.66% came in with multiple injuries. GCS scores were available for 163 patients, and ASA scores were available for 160 patients. The mean (±SD) GSC was 14.04 ± 2.57 in men and 14.74 ± 1.46 in women (*p* = 0.03; 95% CI [−1.33; −0.07]). The ASA Physical Status score [10] was 2.26 ± 0.99 in men and 2.49 ± 1.01 in women (*p* = 0.152; 95% CI [−0.54; 0.09]). Sixteen patients had type 2 diabetes mellitus, and two patients had type 1 diabetes mellitus (Table 1).

Analysis of the Abbreviated Injury Scale (AIS) yielded the following results. The mean AIS score for the spine was 2.83 ± 0.72 in men (n = 93) and 2.62 ± 0.59 in women (n = 71). In men, the mean AIS scores were 1.78 ± 1.10 for the cervical spine, 1.87 ± 0.88 for the thoracic spine, and 1.47 ± 0.8 for the lumbar spine. In women, the mean AIS scores were 1.41 ± 0.87 for the cervical spine, 1.62 ± 0.83 for the thoracic spine, and 1.76 ± 0.76 for the lumbar spine. The difference in overall spinal AIS between men and women was statistically significant (*p* = 0.049, 95% CI [0.01; 0.42]). Significant differences were also observed in the cervical and lumbar regions (cervical: *p* = 0.015; 95% CI [0.07; 0.68] and lumbar: *p* = 0.022; 95% CI [−0.53; −0.04]). No statistically significant difference was observed for the thoracic spine (*p* = 0.065; 95% CI [−0.02; 0.52]). The injury severity score (ISS) could not be analyzed due to insufficient sample size (n = 9). The mean ± SD body mass index (BMI) for men was 25.19 ± 3.54, and for women, it was 24.78 ± 4.95, with no statistically significant difference (*p* = 0.568; 95% CI [−1.01; 1.82]) (Table 2).

Serum electrolyte analysis yielded the following results. Calcium levels were 2.16 ± 0.24 mmol/L in men (n = 68) and 2.19 ± 0.14 mmol/L in women (n = 59), with no statistically significant difference (*p* = 0.275; 95% CI [−0.11; 0.03]). Sodium levels were 136.27 ± 9.91 mmol/L in men (n = 90) and 137.75 ± 3.95 mmol/L in women (n = 71), with no statistically significant difference (*p* = 0.238; 95% CI [−3.94; 0.98]). Potassium levels were 3.91 ± 0.44 mmol/L in men (n = 88) and 3.87 ± 0.48 mmol/L in women (n = 69), with no statistically significant difference between sexes (*p* = 0.59; 95% CI −0.11; 0.19]) (Table 3).

Furthermore, complete blood count parameters were analyzed, including the white blood cell count (WBC, [G/L]), hemoglobin (Hb, [g/L]), and Hematocrit (Hct, [%]). WBC values were 7.21 ± 1.98 G/L in men (n = 88) and 7.35 ± 2.05 G/L in women (n = 69), with no statistically significant difference (*p* = 0.67; 95% CI [−0.80; 0.52]). Hematocrit (Hct) was 0.38 ± 0.06 in men (n = 91) and 0.36 ± 0.05 in women (n = 71), with a statistically significant difference between sexes (*p* = 0.034; 95% CI [0.001; 0.036]). Hemoglobin (Hb) levels were 126.93 ± 20.22 g/L in men (n = 91) and 118.59 ± 18.37 g/L in women (n = 71). This difference was statistically significant (*p* = 0.007; 95% CI [2.26; 14.42]). Additional C-reactive protein (CRP) measurements were analyzed, which displayed no statistical significance (*p* = 0.787; 95% CI [−8.95; 11.78]) (Table 3).

### 3.2. Spine-Specific Data

The intact vertebra above the fracture showed a mean HU value of 55.8 ± 11.2 in men (n = 88) and 56.4 ± 11.9 in women (n = 69). The difference between men and women was statistically not significant (*p* = 0.74; 95% CI [−4.1; 2.9]). The mean HU values for the intact vertebra below the fracture were 42.6 ± 9.8 in men and 41.9 ± 10.5 in women (Table 4).

No statistically significant difference was observed (*p* = 0.67; 95% CI [−2.5; 3.9]). Both HU values of the vertebra above the fracture, as well as HU values below the fracture, showed no significant difference between men and women (Table 5).

Four different surgical treatment options were observed in this cohort for patients with traumatic vertebral fractures. Some patients received more than one treatment. Spinal fusions were performed in 102 patients (63 men, 39 women), representing 60.4% of the cases. Vertebroplasty was performed in 31 patients (15 men, 16 women), accounting for 18.3% of cases. Kyphoplasty was performed in 61 patients (32 men, 29 women), representing 36.1% of cases. The most frequently performed procedure was cement augmentation, which was performed in 147 patients (87% of cases) (Table 6).

The association between surgical procedures and sex was evaluated using chi-square tests—specifically, the Pearson chi-square test—continuity-corrected chi-square, likelihood ratio, and linear-by-linear association, as well as Fisher’s exact test. All tests showed no statistically significant association between sex and surgical procedures. The Pearson chi-square test yielded *p* = 0.602, the continuity-corrected chi-square test *p* = 0.847, the likelihood ratio *p* = 0.605, the linear-by-linear association *p* = 0.604, and Fisher’s exact test *p* = 0.745 (Table 7).

Intervertebral angles were categorized as “preoperative”, “postoperative”, and “one-year postoperative” (Table 8).

The mean preoperative angle was 11.20° ± 12.88° in men (n = 82) and 10.91° ± 7.92° in women (n = 58), with no statistically significant difference (*p* = 0.88; 95% CI [−3.48; 4.06]). The mean postoperative angle was 10.91° ± 10.84° in men (n = 85) and 11.34° ± 7.11° in women (n = 66), also showing no statistically significant difference (*p* = 0.783; 95% CI [−3.47; 2.62]). At one-year follow-up, the mean angle was 8.87° ± 6.85° in men (n = 67) and 13.11° ± 9.33° in women (n = 58), showing a statistically significant difference (*p* = 0.004; 95% CI [−7.12; −1.37]). The Bayesian analysis showed no statistically significant differences for the preoperative angle (95% CI [−3.79; 3.22]) and postoperative angle (95% CI [−2.49; 3.34]). In contrast, the one-year postoperative angle demonstrated a significant difference between sexes (95% CI [1.27; 7.21]) (Table 9).

For a more specific analysis of the surgical outcome, the difference (∆) between the postoperative angle and the one-year postoperative angle was conducted. The results revealed that the ∆ had a *p*-value of 0.094 and a 95% CI of [−0.27; 3.36], indicating no statistically significant difference in sexes. To further inspect the ∆, a statistic regarding the effect sizes was added. Effect sizes were small (Cohen’s d (=0.304), Hedges’ g (=0.302), and Glass’s ∆ (=0.267)) (Table 10).

## 4. Discussion

The retrospective cohort study, including 164 patients, identified statistically significant differences between males and females regarding the age distribution, GCS score, and AIS score of the overall spine, as well as the cervical and lumbar regions, hemoglobin and hematocrit, the Hounsfield unit (HU) of the intact vertebra above the fracture, and the intervertebral angle after one year postoperative. No statistically significant differences were observed in the ASA number, AIS of the thoracic region, body mass index (BMI), or the serum electrolytes, such as calcium, sodium, and potassium, along with surgical treatment options and pre/postoperative intervertebral angles between sexes.

The study showed that women were, on average, 6.46 years older than men at the time of diagnosis. A retrospective cohort study published in the *Journal of Orthopaedia Surgery and Research* reported that male patients were significantly younger when sustaining traumatic vertebral fractures, with a median age of 56 years (IQR 39–70). This difference may be explained by the study design, as this study focused on traumatic vertebral fractures and not osteoporotic fractures, which are more likely to present in female patients [13].

Among men, 61.29% of patients with vertebral fractures presented with polytrauma at the time of admission to the Department of Traumatology. A study published in *The Bone & Joint Journal* reported that major trauma patients were predominantly male with a lower mean age than non-major trauma cases [14]. Our findings are consistent with these results, since polytraumas were more common in male patients (43.66% of women suffered a polytrauma), and they had a significantly lower GCS scores upon hospital admission than women.

The AIS for cervical and lumbar vertebral fractures showed significantly higher scores in men than in women. A study conducted in the Chinese cities Chongqing and Shenyang reported that lumbar and cervical vertebral fractures were more common than thoracic fractures [15]. Another study found significantly higher mean injury severity scores (ISSs) concerning cervical spinal fractures and non-spinal injuries in male patients [16]. German researchers reported a higher proportion of male patients sustaining AO type C fractures—although it is unclear if the difference in sexes was significant [17].

Even though two studies were conducted in Asia and one in Europe, our regional registry-based study showed very similar findings. These findings support our results indication that men have a significantly higher AIS spine score than women, while also mentioning that cervical fractures were significantly more severe in men. Although it is not possible to transfer AO and ISS classification into AIS classification, it may be cautiously suggested that there is a higher ISS for cervical fractures in men [15], and the increase in type C AO fractures in male patients [17] might show a connection between our significant gender difference in the AIS score. However, it is necessary to mention that this claim needs further research to be fully supported.

Our data showed significant differences between men and women in hemoglobin and hematocrit levels at admission. A level I trauma center in Germany found that admission hemoglobin levels of women were significantly lower than in men (10.3 ± 2.4 vs. 11.9 ± 2.1) and suggested that this may contribute to a greater physiological burden in women [18]. A retrospective cohort study from the University Hospital Zurich found that women had a significantly lower hematocrit level (32% vs. 36%) at hospital admission than men [19]. A Korean study researched the connection between low hemoglobin and bone density; they reported an association between these variables, but further analysis is necessary [20]. However, this association has not yet been consistently demonstrated as statistically significant. Conceivable reasons for a hemoglobin and hematocrit difference between men and women could be explained by hormonal factors. Men have higher levels of androgens in their bodies, whereas estrogen predominates in women. Testosterone is known to stimulate erythropoiesis in the red bone marrow. W. P. Murphy [21] published a paper in 2014 writing about how estrogen dilates the renal microvasculature and, therefore, decreases the hematocrit, whereas in men, the androgens constrict such microvasculature and thereby influence erythropoietin regulation.

The Hounsfield unit (HU) has been proposed as a potential indicator for early treatment selection. However, our data provided no statistically significant difference in the HU value of the vertebra above or below the affected vertebra between men and women. These findings suggest that HU measurements in this cohort are likely not influenced by sex. Nguyen et al. [22] published a paper highlighting the clinical relevance of HU values. They identified an association between poor bone quality and instrumentation failure. Our data showed that the one-year postoperative intervertebral angle in women was larger than the immediate postoperative angle, resulting in a significant difference compared to men (*p* = 0.004; 95% CI [−7.12; −1.37])—although the ∆ between the intervertebral angles from postoperative measurements compared to one-year postoperative measurements provided no statistical significance. Schreiber et al. [23] conducted similar findings to Nguyen et al. [22] and mentioned that certain risk assessments and early initiation of treatment planning could benefit from this information. This suggests that the difference develops over a period after surgery, rather than being caused by the initial surgical procedure. This difference may develop over time due to factors such as hormonal influences, degeneration, axial loading, or muscle strength. The reason why the HU value of the vertebra below the fracture did not show significance remains unclear. There are two possible options that could explain this. Firstly, a statistical artifact cannot be excluded. Secondly, the axial loading may be greater on the cranial vertebra, therefore being stressed more and showing degenerative signs earlier.

Biological differences in bone density should still be considered. Estrogen in women is primarily responsible for bone protection. After menopause, estrogen levels decrease, leading to an increased incidence of osteoporosis. While this could theoretically influence HU values, our study did not detect a corresponding difference between sexes. However, the *European Spine Journal* published a paper in 2025, providing results that showed a significant connection between the decreased HU value and postmenopausal women [24].

Although several variables showed significant differences between men and women, many did not demonstrate any association, such as BMI, ASA number, or serum electrolytes, like calcium, sodium, and potassium. This may be due to the relatively small sample size.

Aside from our data not finding a significant association with BMI, a Japanese cohort study showed that a BMI < 18.5 kg/m^2^ is associated with an increased risk for vertebral fractures only in men [25]. This may be explained by decreased estrogen levels associated with low body fat, since fatty tissue contains aromatase, which is needed for the conversion of testosterone into estrogen.

Limitations of this study are the fact that it is a retrospective data collection based on a single regional registry, which may introduce information and selection bias, as well as limited generalizability, especially since only patients who underwent surgical treatment were included. The data also lacks functional outcomes and missing adjustments for confounders, such as age. To minimize bias, standardized procedures were used when extracting electronic medical records, and strict inclusion criteria were applied. Although HU and intervertebral measurements were done with caution, deviations and inter-observer bias can occur. HU values are not a standard method for assessing bone density or diagnosing osteoporosis. Due to some patients not attending follow-up appointments, outcome data was limited. Our data do not allow conclusions about long-term functional outcomes. The sample size for surgical treatment options was small, therefore limiting the statistical power for further subgroup analyses. Furthermore, we were unable to determine whether a fracture was solely of traumatic origin or combined with an underlying osteoporotic cause. Given the number of statistical comparisons performed, no correction method for multiple testing was applied, therefore causing an increased risk of type I error inflation. Future research should include multicenter data with a larger sample size to reduce the likelihood of bias.

The strengths of this study include a relatively large number of patients sampled from a single hospital during only one year. This study included direct clinical data, extensive CT imaging, and clear definitions.

These findings may contribute to future individual planning and perioperative management. In conclusion, the findings in this paper highlight the importance of considering sex-specific factors for treatment discussion.

## 5. Conclusions

Sex-specific factors may improve diagnostic accuracy and support more individualized treatment planning. This study provides insight into the biological and mechanical differences between men and women in traumatic vertebral fractures. Hounsfield unit (HU) values may be useful for preoperative assessment and treatment selection, potentially reducing the risk of long-term complications and highlighting the need for further research. In cases of suspected osteopenia or osteoporosis, preventive therapy with Teriparatide [26] may be considered as part of an individualized treatment approach, particularly in women. In surgical management, the use of methyl methacrylate (MMA) may be considered in selected cases.

## Figures and Tables

**Table 1:** Patient sample description.

Sex		Age	GCS	Single Trauma	Polytrauma	ASA
**Male**	***N***	93	93	93	93	91
	***mean***	55.24	14.04	0.39	0.61	2.26
	***SD***	20.59	2.57	0.05	0.49	0.99
**Female**	***N***	71	70	71	71	69
	***mean***	61.69	14.74	0.56	0.44	2.49
	***SD***	18.75	1.46	0.056	0.49	1.01
**Total**	***N***	164				
	***mean***	58.03				
	***SD***	20.02				
**Significance**		0.04	0.03			0.15

**Table 2:** Gender distribution of AIS of the complete spine, cervical/thoracic, and lumbar sections, as well as BMI.

Sex		AIS Spine	AIS Cervical	AIS Thoracic	AIS Lumbar	BMI
**Male**	***N***	93	93	93	93	87
	***mean***	2.83	1.78	1.87	1.47	25.19
	***SD***	0.72	1.09	0.88	0.80	3.54
**Female**	***N***	71	71	71	71	67
	***mean***	2.62	1.41	1.62	1.76	24.78
	***SD***	0.59	0.87	0.83	0.76	4.95
**Significance**		0.049	0.02	0.07	0.02	0.57

**Table 3:** Patient sample description.

Sex		Ca^2+^	Na^+^	K^+^	WBC	Hct	Hb
**Male**	***N***	68	90	88	88	91	91
	***mean***	2.16	136.27	3.91	7.21	0.38	126.93
	***SD***	0.24	9.91	0.44	1.98	0.06	20.22
**Female**	***N***	59	71	69	69	71	71
	***mean***	2.20	137.75	3.87	7.35	0.36	118.58
	***SD***	0.14	3.95	0.48	2.05	0.05	18.37
**Significance**		0.28	0.24	0.59	0.67	0.03	0.01

**Table 4:** Gender distribution of the HU values.

	Sex	N	Mean	SD	SEM
**HU above**	male	88	55.8	11.2	1.19
	female	69	56.4	11.9	1.43
**HU below**	male	88	42.6	9.8	1.04
	female	69	41.9	10.5	1.26

**Table 5:** Levene test and significance in the gender difference of HU values.

		Levene Test	Levene Test	*t*-Test
		F	Sig.	2-Sided *p*
**HU above**	Variance same	0.41	0.52	0.74
	Variance different			0.74
**HU below**	Variance same	0.32	0.57	0.67
	Variance different			0.67

**Table 6:** Distribution of surgical procedures.

Surgical Treatment	N	%
**spinal fusion**	102	60.4
**vertebroplasty**	31	18.3
**kyphoplasty**	61	36.1
**cement augmentation**	147	87

**Table 7:** Significance levels of gender distributions on surgical options.

	*p*-Value
**Pearson chi-square**	0.602
**Continuity correction**	0.847
**Likelihood ratio**	0.605
**Fisher’s exact test**	0.745
**Linear-by-linear association**	0.604

**Table 8:** Evaluation of intervertebral angles.

	Sex	N	Mean	SD
**preoperative angle**	male	82	11.20	12.88
	female	58	10.91	7.92
**postoperative angle**	male	85	10.91	10.85
	female	66	11.34	7.11
**one-year postoperative angle**	male	67	8.87	6.85
	female	58	13.11	9.33

**Table 9:** Significances of gender distributions on intervertebral angles.

		*p*-Value
**preoperative angle**	Var. same	0.88
	Var. different	0.87
**postoperative angle**	Var. same	783
	Var. different	0.77
**one-year postoperative angle**	Var. same	0.004
	Var. different	0.005

**Table 10:** Effect sizes of intervertebral angle differences between genders.

		Point Estimate
**∆ 1 year**	Cohen’s d	0.304
	Hedges’ g	0.302
	Glass’ delta	0.267

## Data Availability

The data presented in this study are not publicly available due to ethical and privacy restrictions related to patient confidentiality.

## Abbreviations

The following abbreviations are used in this manuscript:

AO	Arbeitsgemeinschaft für Osteosynthesefragen
DEXA	Dual-energy X
MMA	Methyl methacrylate
BMI	Body mass index
IBM SPSS	IBM Statistical Package for the Social Sciences
CRP	C-reactive protein
GCS	Glasgow Coma Scale
ASA	American Society of Anesthesiologists
ISS	Injury severity score
AIS	Abbreviated injury scale
CT	Computed tomography
HUs	Hounsfield units
SEM	Standard error of the mean
CI	Confidence interval
SD	Standard deviation
WBC	White blood cell count
Hb	Hemoglobin
Hct	Hematocrit
K^+^	Potassium
Ca^2+^	Calcium
Na^+^	Sodium
Var.	Variance
